# Supplementary material for: Feasibility of smartphone-based digital phenotyping to measure visual function and mental health outcomes in patients with inherited retinal diseases
Source: NPJ Digit Med. 2026 May 29;9:601. doi: 10.1038/s41746-026-02817-w (PMC13442882; doi:10.1038/s41746-026-02817-w)
Supplement: Supplementary file 1 [file 41746_2026_2817_MOESM1_ESM.pdf]

Supplementary Table 1

STROBE Statement—checklist of items that should be included in reports of observational studies

	Item No.	Recommendation	Page No.	Relevant text from manuscript
**Title and abstract**	1	(*a*) Indicate the study's design with a commonly used term in the title or the abstract	Title (p1)<br><br>Introduction (p4)	p4: "This pilot feasibility study evaluated the use of a bespoke smartphone application (*OverSight*) designed to capture passive and active digital phenotyping data in individuals with IRDs".
		(*b*) Provide in the abstract an informative and balanced summary of what was done and what was found	Abstract (p2)	"This study evaluated the feasibility of collecting passive and active digital phenotyping data using the *OverSight* iOS application in individuals with inherited retinal diseases… Twenty-five participants were enrolled and 92% were retained at 12 months… Digital phenotyping using *OverSight* was feasible over 12 months."
**Introduction**				
Background/rationale	2	Explain the scientific background and rationale for the investigation being reported	Introduction (p3)	p3: "Inherited retinal diseases (IRDs) are a heterogeneous group of genetic eye disorders… Visual impairment arising from IRDs can substantially affect everyday

				functioning… Digital phenotyping incorporates passive data and patient-reported outcomes to capture real-world functional and mental health status.”
Objectives	3	State specific objectives, including any prespecified hypotheses	Introduction (p4)	p4: “This pilot feasibility study evaluated the use of a bespoke smartphone application (*OverSight*)… A secondary exploratory objective was to examine associations between digital behavioural metrics, self-reported visual functioning, and mental health indicators.”
**Methods**				
Study design	4	Present key elements of study design early in the paper	Methods (p11)	p11: “Design: This pilot feasibility study involved onboarding individuals with RP and CHM to the *OverSight* smartphone app to assess enrolment, retention, and completeness of passive and active digital phenotyping datastreams.”
Setting	5	Describe the setting, locations, and relevant dates, including periods of recruitment, exposure, follow-up, and data collection	Methods (p12 – p13)	p12: “Participants were recruited from the Genetic Eye Disease Service at Moorfields Eye Hospital NHS Foundation Trust.”

				p13: "Passive datastreams… were collected over 12 months (March 2023–February 2024)."
Participants	6	(*a*) *Cohort study*—Give the eligibility criteria, and the sources and methods of selection of participants. Describe methods of follow-up<br>*Case-control study*—Give the eligibility criteria, and the sources and methods of case ascertainment and control selection. Give the rationale for the choice of cases and controls<br>*Cross-sectional study*—Give the eligibility criteria, and the sources and methods of selection of participants	Methods (p12)	p12: "Eligible participants were aged 16 years or older, had a molecularly confirmed diagnosis of RP or CHM, and a better eye VA of 6/60 Snellen (<1.0 logMAR) or better."<br><br>p12: "Opportunity sampling was used, with all eligible patients invited to participate."
		(*b*) *Cohort study*—For matched studies, give matching criteria and number of exposed and unexposed<br>*Case-control study*—For matched studies, give matching criteria and the number of controls per case	N/A	N/A
Variables	7	Clearly define all outcomes, exposures, predictors, potential confounders, and effect modifiers. Give diagnostic criteria, if applicable	Methods (p13)	p13: "The primary outcome was feasibility of collecting passive and active digital phenotyping data… Secondary outcomes explored associations between passive digital metrics and participant characteristics and PROMs."
Data sources/ measurement	8*	For each variable of interest, give sources of data and details of methods of assessment (measurement). Describe comparability of assessment methods if there is more than one group	Methods (p12)	p12: "*OverSight* was developed using Apple HealthKit and SensorKit frameworks. HealthKit provides aggregated physical activity and mobility data… SensorKit enables access

				to system-level keyboard metrics.”
Bias	9	Describe any efforts to address potential sources of bias	Discussion / limitations (p10)	p10: “The small sample size limits generalisability… recruitment from a single specialist centre may have introduced response bias… variability in smartphone use and device type likely contributed to noise within passive datastreams.”
Study size	10	Explain how the study size was arrived at	Methods (p12)	p12: “As this study was designed as a pilot feasibility evaluation, no formal sample size calculation was performed. The sample size was determined pragmatically based on the number of eligible patients available during the recruitment period.”

Continued on next page

Quantitative variables	11	Explain how quantitative variables were handled in the analyses. If applicable, describe which groupings were chosen and why	Methods (p13)	p13: "Daily step and walking speed values were aggregated into weekly averages… A valid day was defined as ≥100 recorded steps, and a valid month as ≥25 valid days."
Statistical methods	12	(*a*) Describe all statistical methods, including those used to control for confounding	Methods (p14)	p14: "Spearman's rank correlation coefficients were calculated to assess associations… Multiple comparisons were corrected using the Benjamini–Hochberg procedure."
		(*b*) Describe any methods used to examine subgroups and interactions	Methods (p14)	p14: "Exploratory analyses examined associations between passive datastreams and participant characteristics and PROMs."
		(*c*) Explain how missing data were addressed	Methods (p14)	p14: "Feasibility was further evaluated based on the number of participants meeting predefined data quality thresholds: at least six months of high-fidelity mobility data from HealthKit and a minimum of three days of keyboard data from SensorKit."
		(*d*) *Cohort study*—If applicable, explain how loss to follow-up was addressed<br>*Case-control study*—If applicable, explain how matching of cases and controls was addressed<br>*Cross-sectional study*—If applicable, describe analytical methods taking account of sampling strategy	Results (p5)	p5: "Study retention over 12-month was 92% (23 out of 25), with attrition occurring only within the first 3 months and no subsequent withdrawals."
		(*e*) Describe any sensitivity analyses	N/A	N/A
**Results**				
Participants	13*	(a) Report numbers of individuals at each stage of study—eg numbers potentially eligible, examined	Figure 1 &	Figure 1. Flow chart

		for eligibility, confirmed eligible, included in the study, completing follow-up, and analysed	Results (p5)	p5: "All 25 enrolled participants successfully downloaded the OverSight app and completed onboarding. Study retention over 12-month was 92% (23 out of 25)…Of the 17 participants with valid HealthKit data, 94% (n = 16) also met the SensorKit keyboard data threshold".
		(b) Give reasons for non-participation at each stage	Figure 1	"Figure 1. Participant flow and data completeness across the 12-month feasibility study: Of 50 individuals pre-screened, 25 were enrolled, with exclusions primarily due to incompatible smartphones or declining to participate. Retention at 12 months was 92% (n = 23). HealthKit data were analysable for 17 participants, with missingness attributable to device-related technical issues rather than participant withdrawal."
		(c) Consider use of a flow diagram	Figure 1	Figure 1
Descriptive data	14*	(a) Give characteristics of study participants (eg demographic, clinical, social) and information on exposures and potential confounders	Table 2 & Results (p5)	p5: "The mean age was 39 years… eight males and nine females… mean better eye VA was 0.28 logMAR."
		(b) Indicate number of participants with missing data for each variable of interest	Figure 1	Figure 1
		(c) *Cohort study*—Summarise follow-up time (eg, average and total amount)	Results (p5)	p5: "All 17 participants provided 6 months of valid mobility data

				(HealthKit), collected between May 2023 and October 2023."
Outcome data	15*	*Cohort study*—Report numbers of outcome events or summary measures over time	Results (p5)	p5: "Median weekly step count was 6,087… median walking speed was 1.18 m/s… PROM completion was 74%."
		Case-control study—Report numbers in each exposure category, or summary measures of exposure	N/A	N/A
		Cross-sectional study—Report numbers of outcome events or summary measures	N/A	N/A
Main results	16	(*a*) Give unadjusted estimates and, if applicable, confounder-adjusted estimates and their precision (eg, 95% confidence interval). Make clear which confounders were adjusted for and why they were included	Methods (p14), Results & Figure 2,3 & 4.	p14: "Spearman's rank correlation coefficients were calculated to assess the strength and direction of associations. Multiple comparisons were corrected using the Benjamini–Hochberg procedure."<br><br>p5: "Median (IQR) weekly step count was 6,087 (3,891–8,344), and median (IQR) weekly walking speed was 1.18 m/s (1.08–1.26). Individual trajectories are shown in Figures 2–3."<br><br>p6: "After false discovery rate correction, several associations remained…Age was negatively correlated with typing speed (ρ = –0.79, p-adj = 0.03)…Age also correlated negatively with the average number of anxious-related words typed (ρ = –0.72, p-adj = 0.04) … Photopic peripheral

			function exhibited strong negative correlations with both the total number of anxious-related words and down-related words (ρ = –0.78 for both, p-adj = 0.04) … (see Figure 4).”
	(*b*) Report category boundaries when continuous variables were categorized	N/A	N/A
	(*c*) If relevant, consider translating estimates of relative risk into absolute risk for a meaningful time period	N/A	N/A

Continued on next page

Other analyses	17	Report other analyses done—eg analyses of subgroups and interactions, and sensitivity analyses	Results (p6)	p6: “Several additional associations were present at trend level but did not meet corrected significance thresholds, including contrast sensitivity and typing speed (ρ = −0.69, p = 0.09), younger age and down-related word use (ρ = −0.70, p = 0.06), younger age and total words typed (ρ = −0.67, p = 0.07), and photopic peripheral visual function with total words typed, typing speed, and health-related word use (ρ = −0.69 for all, p = 0.08).”
**Discussion**				
Key results	18	Summarise key results with reference to study objectives	Discussion (p7)	p7: “This pilot study demonstrated the feasibility of using the *OverSight* smartphone app to collect passive and active digital phenotyping data in individuals with IRDs. Among participants retained at 12-months, sustained passive data capture meeting predefined HealthKit quality thresholds was achieved in 74% of participants, and 92% of these also met SensorKit thresholds.”
Limitations	19	Discuss limitations of the study, taking into account sources of potential bias or imprecision. Discuss both direction and magnitude of any potential bias	Discussion (p10-11)	p10: “With respect to limitations, the small sample size, typical of pilot work, limits generalisability…”

Interpretation	20	Give a cautious overall interpretation of results considering objectives, limitations, multiplicity of analyses, results from similar studies, and other relevant evidence	Discussion (p11)	p11: "These findings indicate that smartphone-based collection of mobility, typing, and patient-reported outcomes is practicable in this population. As feasibility outcomes, the results should be interpreted as preliminary."
Generalisability	21	Discuss the generalisability (external validity) of the study results	Discussion (p10-11)	p11: "Sentiment analysis was based on English-language lexical categories, and findings may not generalise to multilingual populations or to individuals who primarily communicate in other languages. This should be considered in future research where multilingual populations are recruited. Lastly, the *OverSight* app was compatible only with Apple iOS devices, which may introduce digital exclusion and limit generalisability."
**Other information**				
Funding	22	Give the source of funding and the role of the funders for the present study and, if applicable, for the original study on which the present article is based	p15	p15: "This study was funded by UKRI Economic and Social Research Council (grant number ES/W006510/1), Wellcome Trust (grant number 205174/Z//16/Z), Thomas Pocklington Trust, and the NIHR Moorfields Biomedical Research Centre. The funder played no role in study design, data

			collection, analysis and interpretation of data, or the writing of this manuscript.”

*Give information separately for cases and controls in case-control studies and, if applicable, for exposed and unexposed groups in cohort and cross-sectional studies.

Note: An Explanation and Elaboration article discusses each checklist item and gives methodological background and published examples of transparent reporting. The STROBE checklist is best used in conjunction with this article (freely available on the Web sites of PLoS Medicine at http://www.plosmedicine.org/, Annals of Internal Medicine at http://www.annals.org/, and Epidemiology at http://www.epidem.com/). Information on the STROBE Initiative is available at www.strobe-statement.org.
